# Calibration and Validation of a Linear-Elastic Numerical Model for Timber Step Joints Based on the Results of Experimental Investigations

**DOI:** 10.3390/ma15051639

**Published:** 2022-02-22

**Authors:** Matthias Braun, Jan Pełczyński, Anna Al Sabouni-Zawadzka, Benjamin Kromoser

**Affiliations:** 1 Institute of Structural Engineering, University of Natural Resources and Life Sciences (BOKU), 1190 Vienna, Austria; benjamin.kromoser@boku.ac.at; 2 Faculty of Civil Engineering, Warsaw University of Technology, 00-637 Warsaw, Poland; j.pelczynski@il.pw.edu.pl (J.P.); a.sabouni@il.pw.edu.pl (A.A.S.-Z.)

**Keywords:** timber–timber joints, single-step joint, double-step joint, resource efficient construction, digital image correlation, finite element analysis

## Abstract

The paper is dedicated to the numerical analysis of a single-step joint, enabling the prediction of stiffness and failure modes of both single- and double-step joints. An experimental analysis of the geometrically simplest version, the single-step joint, serves as a reference for the calibration of the subsequent finite element model. The inhomogeneous and anisotropic properties of solid timber make detailed modelling computationally intensive and strongly dependent on the respective specimen. Therefore, the authors present a strategy for simplified but still appropriate modelling for the prediction of local failure at certain load levels. The used mathematical approach is based on the linear elasticity theory and orthotropic material properties. The finite element calculations are performed in the environment of the software Abaqus FEA. The calibrated numerical model shows a good conformity until first failures occur. It allows for a satisfactory quantification of the stiffness of the connection and estimation of the force when local failure begins and is, therefore, recommended for future, non-destructive research of timber connections of various shapes.

## 1. Introduction

We are currently confronted with a steadily increasing CO_2_ concentration in the atmosphere, resulting in global warming [1]. A large part (~40%) of the global CO_2_ emissions is caused by the construction industry [2]. The comparison of different construction materials shows that timber exhibits advantageous properties with regard to these environmental aspects, as it stores part of the CO_2_ absorbed during the growth phase of the trees [3]. Unfortunately, a scarcity of the raw material has been observed on the present market, resulting in, among other things, a strong price increase. If timber is to be used in larger quantities as a construction material, a higher degree of utilisation is, therefore, essential.

An outstanding example of efficient material use is timber truss systems which have optimal load-bearing behaviour while highly utilising the construction material [4]. Unfortunately, the high effort in design and production make the manufacturing and application of these structures in this day and age a rarity. New, upcoming possibilities, such as digital tools and CNC machines, enable an automation of the design and production process and allow for the improvement of the profitability in order to, yet again, be able to compete with other systems such as plate girders [4]. A key factor in the functionality and efficiency of timber truss systems is the joints.

Previous research by the Institute of Structural Engineering at BOKU showed that timber–timber step joints represent very efficient solutions for the transfer of compressive forces. The joints provide a ductile failure behaviour, are easy to produce and are able to transfer comparatively high loads. In addition, no additional steel parts, which normally have a major impact on the environmental performance and economy of such structures, are required. A series of compression tests performed by the authors, investigating different embodiments of step joints, as well as inaccuracies possibly caused by a change of moisture content or production, showed the influence of the geometry on the performance (stiffness and maximum load) [4,5,6,7].

The authors of this paper pursued two main goals while driving research forward in this area: (1) Experimental investigation of the load-bearing behaviour of new timber step joint designs with a set focus on maximising the performance (maximum load, stiffness and producibility) [6] (BOKU, Institute of Structural Engineering) and (2) Development of a modelling strategy for timber–timber joints under compression for the best possible prediction of the load-bearing behaviour (Warsaw University of Technology, Faculty of Civil Engineering). The basic research approaches to achieve the defined goals were the implementation of experimental investigations on the one side and increasing sophistication of the finite element model on the other.

A realistic prediction of the load-bearing behaviour prior to construction counts as one of the core disciplines of structural engineering. In addition to analytical models and empirical determined data, finite element (FE) calculations can be added to the well-established prediction tools. However, a careful (material) model calibration based on experimental investigations, especially for materials with a complex behaviour, such as timber, is the basis for a reliable prediction model. The main focus of the presented paper was the investigation of the performance and applicability of a simplified FE model based on linear-elastic material behaviour and an orthotropic constitutive model, allowing for a comparatively low computation effort.

After the introduction, the test setup and test specimens, as well as the results of the performed experimental investigations on a single-step joint, are explained in Section 2. Section 3 is dedicated to the mathematical model used to simulate the behaviour of timber within an orthotropic constitutive model of linear elasticity used for the FE simulation performed with the Abaqus FEA software. Subsequently, Section 4 deals with the computational modelling and the calibration of the material parameters based on the results of the experiments. In Section 5, the linear-elastic model is applied to the geometry of a double-step joint and, again, compared with the results of experimental investigations. Concluding remarks can be found in Section 6.

## 2. Experimental Investigations

As a first basis for the FE model calibration, a single-step joint (Series A) was chosen. Compared to joints with two (double-step joints) or a number of steps (multi-step joints) the influence of production inaccuracies is lower within the single-step joint, making it easier to identify the individual influencing parameters. Subsequently, the results of experiments on double-step joints (Series B) were used to verify if the calibration could be applied to other geometries. The experimental investigations analysing the load-bearing behaviour of single- and double-step joints were performed in the laboratory of the Institute of Structural Engineering at BOKU.

### 2.1. Test Setup and Test Specimens

**Figure 1:**
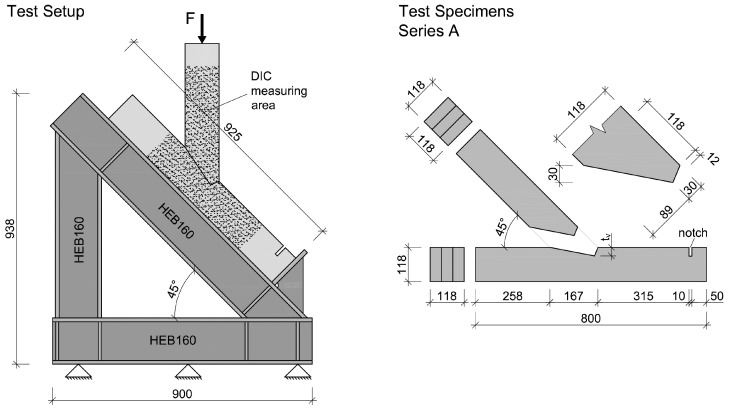
(**Left**): Test setup [4]; (**Right**): Geometry of the Series A test specimens. Measurements in mm.

The test setup, consisting of welded HEB160 steel profiles, as well as the geometry of the test specimens (single-step joint), are pictured in Figure 1. The load was applied using a three-axial, servo-hydraulic testing machine in a displacement-controlled manner with a rate of 1 mm/min. A digital image correlation system (DIC-3D™ from the company Correlated Solutions, Irmo, SC, USA) was used to measure the occurring strains on one surface of the specimens while the loads were recorded using an electric load cell. The areas of interest of the timber specimens were painted white and, subsequently, sprinkled using black paint, creating appropriate reference points. Five tests were performed.

Laminated timber with a strength class GL24h according to ÖNORM EN 14080:2013 [8] and a cross-section of 118 × 118 mm² made of three spruce lamellas was chosen for the specimens. The lamellas were arranged vertically to minimise the influence of local wood defects. The moisture content, varying from 7.3% to 8.9%, was determined using a GANN Hydromette BL H40/HT70 immediately after testing of each specimen. A sufficient length of the shearing path was chosen to create failure by crushing due to compression stress and prevent shear failure. A 30 mm deep notch was cut into the specimens 315 mm from the joint in order to eliminate any influence of the abutment.

### 2.2. Results of the Experimental Invesitations

**Figure 2:**
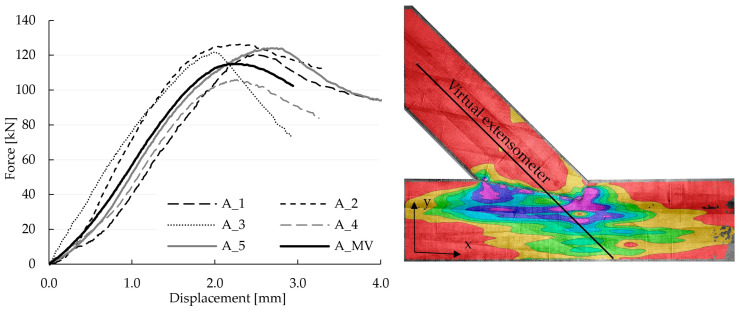
(**Left**): Force-displacement curves of all five test specimens of the single-step joints, as well as the mean value MV of the five specimens; (**Right**): Normal strains eyy at 90% of the ultimate load (124.08 kN) of specimen A_5 and visualisation of the virtual extensometer (based on the DIC measurement) used for the determination of the displacement, as well as the orientation of the coordinate system.

The test results of the load-bearing investigations of the five single-step joints of Series A are summarised in Figure 2 (left). A virtual extensometer, shown in Figure 2 (right), was used during post-processing for the determination of the displacement within the evaluation of the tests using the software VIC-3D 9™. The results clearly show that all specimens exhibited an almost linear-elastic behaviour throughout a specific phase of the experiments. When evaluating the mean of all five specimens (A_MV), the linear-elastic phase extended from 19% to 72% of the achieved ultimate force F_max_. Once the failure occurred in a ductile manner, the tests were stopped, and the post-fractural behaviour was observed. For the calibration of the numerical model, it was necessary to obtain data from one individual specimen because, within VIC-3D 9™, it was not possible to create mean values for points at the exact same location (seen in Section 4.2) for individual specimens. Therefore, the results of one individual specimen were used. As the results of specimen A_5, with the linear-elastic phase ranging from 24 to 82 kN, represented the mean value of this test series best (see Figure 2 left), it was chosen for the calibration. 

**Figure 3:**
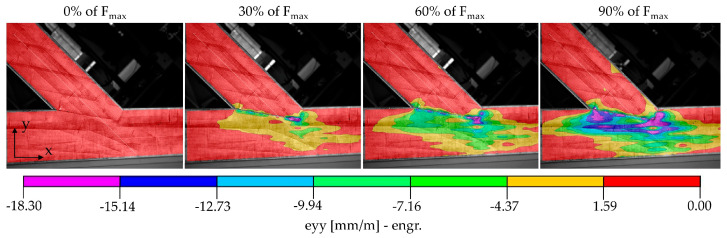
Normal strains eyy of the surface of specimen A_5 measured using the DIC system at 0%, 30%, 60% and 90% of the ultimate load F_max_ at 124.08 kN.

The DIC system measures displacements of reference points on the surface of the specimen, with all other quantities post-processed mathematically. In Figure 3, the normal strains eyy (in vertical direction) of the surface of specimen A_5 at different load levels are shown to illustrate the propagation with increasing load.

**Figure 4:**
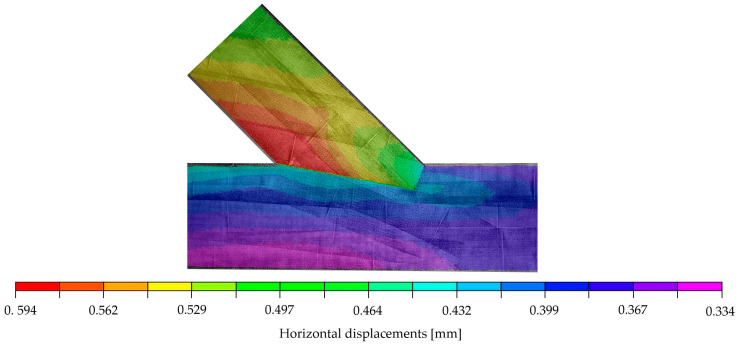
DIC-horizontal displacements on the surface of specimen A_5 at 70.1 kN.

**Figure 5:**
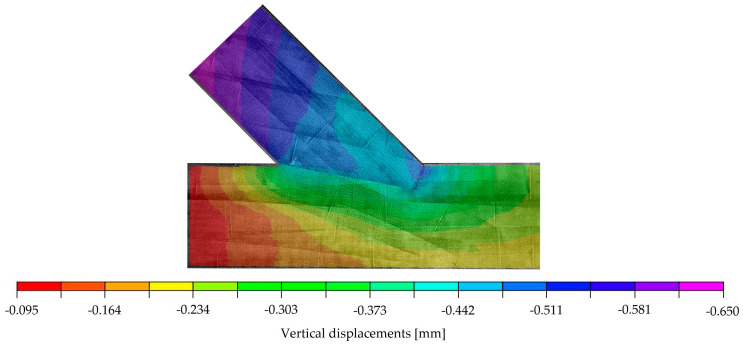
DIC-vertical displacements on the surface of specimen A_5 at 70.1 kN.

The validation of the subsequently proposed numerical model was performed relying on the displacements. Figure 4 and Figure 5 show the distributions of displacements measured with the DIC system for the chosen specimen A_5 at a load of 70.1 kN. This load was set as the top calibration range for the numerical model and represents approximately 85% of the end of the elastic phase. The figures show that, in addition to the deformations of the specimen itself, vertical and horizontal displacements at the lower edge can be traced back to deflections of the steel frame. The results from the virtual extensometer, however, indicate that these deflections of the test setup did not affect the results seen in the force-displacement curves presented in Figure 2 (left).

## 3. Mathematical Model of Timber with the Finite Element Implementation

Various experimental tests of timber joints show a strong dependence of the results on the inhomogeneous material properties of the sample, in particular the arrangement and twist of the fibres, as well as the presence of knots [9,10]. Furthermore, the results are dependent on the chosen geometry of the joint and the accuracy of craftsmanship in regard to the execution [7]. A precise prediction of the load-bearing behaviour considering the inhomogeneity of the material, as well as the damage and the accompanying redistribution of forces in certain areas, requires a detailed analysis of the properties of each sample. A very detailed modelling and subsequent numerical analysis in terms of non-linear-elastic, elastic-plastic, visco-plastic or fracture mechanics are, therefore, necessary to identify the propagation of the failure. Another approach is to use novel hysteretic models, which are capable of simulating the complex, non-linear behaviour of materials [11,12,13]. In order to obtain a first assessment of a joint without complicated calculations, the authors proposed to simplify the numerical analysis to a linear-elastic model. Subsequently, it was investigated whether the simplified model allowed for an assessment of the local failure modes, an estimation of the stiffness of the connection (force-displacement relationship) and a prediction of the load at which the onset of failure is to be expected. 

In a linear-elastic model, the constitutive relations have the following form [14]:

(1) $$S_{ij}=D_{ijlk}E_{kl}$$

with $S_{ij}$ being the components of the stress tensor, $E_{kl}$ the components of the strain tensor and $D_{ijlk}$ the components of the elasticity tensor. In the orthotropic model, non-zero components of elasticity tensor can be expressed by nine independent technical coefficients [14]: three Young’s moduli $E_{1},E_{2},E_{3}$, three Kirchhoff’s moduli $G_{12},G_{13},G_{23}$ and three independent Poisson’s ratios selected from six: $\nu_{12},\nu_{13},\nu_{23},\nu_{21},\nu_{31},\nu_{32}$, with dependencies as follows:

(2) $$\frac{\nu_{12}}{E_{2}}=\frac{\nu_{21}}{E_{1}},\frac{\nu_{13}}{E_{3}}=\frac{\nu_{31}}{E_{1}},\frac{\nu_{23}}{E_{3}}=\frac{\nu_{32}}{E_{2}}$$

The directions are defined as parallel to the grain (direction 1), perpendicular to the grain (direction 2) and circumferential (direction 3). The technical coefficients are usually determined based on experimental research published in literature or taken from the design standards [15]. In the case of the presented model, the technical coefficients, listed in Section 4.1, were calibrated based on the previously described experimental research.

A theoretical analysis of timber joints within the framework of the theory of elasticity is possible with the use of FE modelling (e.g., [16,17,18]). Even though only a certain range of the structural response to the load (linear-elastic phase) is covered by this type of modelling, it is expected that the linear range allows for the determination of the onset of local failure and failure mode. As described in [19], 3D modelling is not always necessary, with 2D models often being sufficient for orthotropic materials [20,21,22], resulting in simulation time reduction and, therefore, a cost reduction within the assessment process. Since no significant, out-of-plane deformations were observed during the experimental investigations, the use of a 2D model for the presented numerical calculations seemed justified.

A detailed comparison of the modelling results with the results of the experimental investigations is presented in the following sections.

## 4. Numerical Calculations

### 4.1. First Step—Numerical Simulation Using Material Properties According to EN 14080

The simulation, based on a geometrical model with dimensions of specimen A_5 (Figure 1) was carried out in Abaqus FEA software. The model itself consisted of two unconnected parts, namely the upper and lower beam. The contact zone of both beams was modelled in terms of unilateral constraints that activate as soon as the elements are pressed against each other, with the possibility of sliding with friction, in accordance with the procedures of Abaqus [23]. In the contact area, surface-to-surface contact was assumed in the initial step, with the option *Adjust only to remove overclosure*. The contact interaction property included *“Hard” Contact Normal behaviour*, with permission for separation after contact and *Tangential behaviour* with the friction coefficient μ. With this model, the contact areas could be calculated frictionlessly when μ=0. 

**Figure 6:**
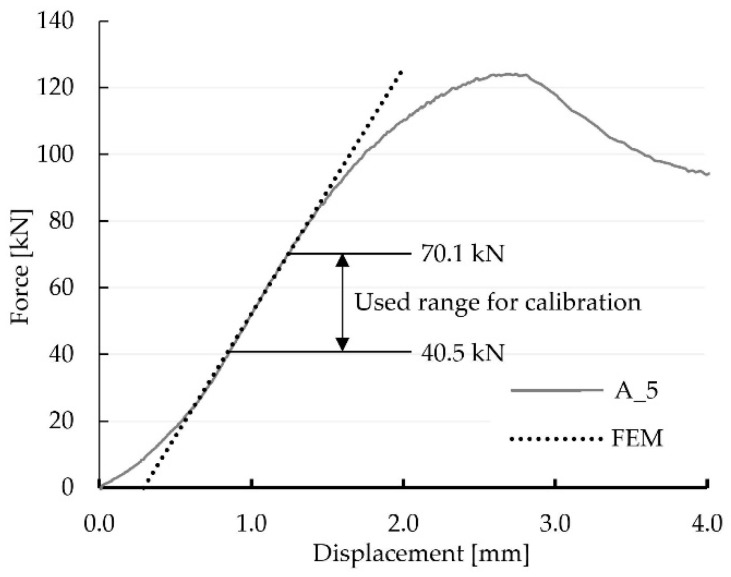
Experimentally, as well as numerically (FEM), determined load-displacement curves of specimen A_5. The FEM line was shifted to the right to facilitate comparability.

**Figure 7:**
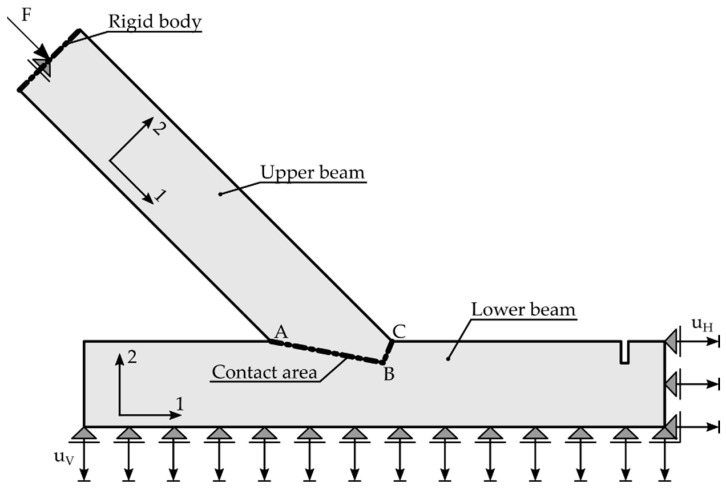
Geometry and designations of the FEM model for the single-step joint. Axis 1 represents the orientation parallel to the grain.

As a linear-elastic approach was chosen to model the experiment, a 29.6 kN increment between 40.5 kN and 70.1 kN was chosen according to the linear-elastic phase of the results of specimen A_5 (see Figure 6). The increment value was based on the choice of the authors to work within the linear-elastic phase in combination with the available data from the DIC measurements. The resulting linear-elastic model allowed for easy scaling of the results and, therefore, an easy comparison with experimental results taken from different load ranges. In the area of the force application, a rigid body, loaded with a concentrated force F=29.6 kN, modelled the behaviour of the testing machine. A vertical displacement $u_{V}=0.211\;\mathrm{mm}$ was imposed at the bottom edge of the lower beam, while a horizontal displacement $u_{H}=0.361\;\mathrm{mm}$ was assumed at the right edge to account for the displacements of the steel frame during the experimental investigations within the investigated load range. The geometrical data and the designation of the contact areas, as well as the boundary conditions for the supports, are pictured in Figure 7.

For the 2D plane stress model, with a thickness of 118 mm, 124,538 square elements with linear shape functions and reduced integration (CPS4R) were used. Additionally, 249 triangular elements CPS3 were applied in the areas of less regular mesh. The total number of Gauss points in the model was 124,787. The mesh size of the finite elements was set to approximately 3 mm in general areas and 1 mm close to the contact area in order to provide higher precision of the results. The mesh was generated by Abaqus algorithm with seeding conditions provided by the authors.

**Figure 8:**
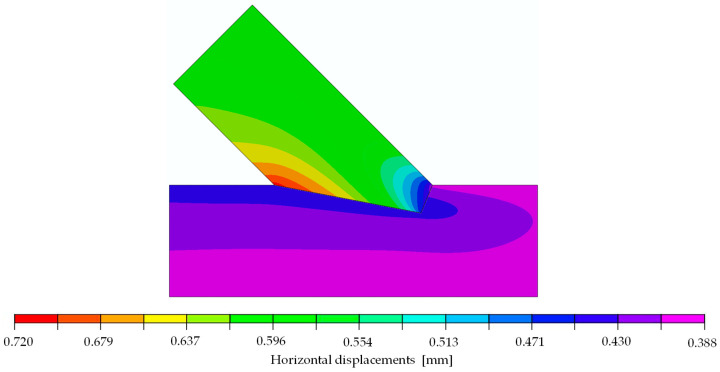
Horizontal displacements of numerical model at 29.6 kN [mm].

**Figure 9:**
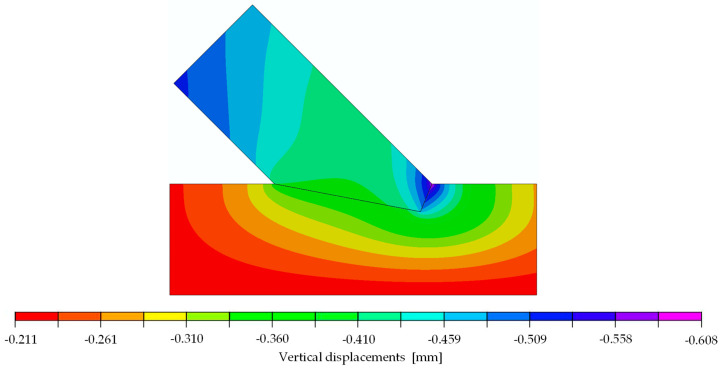
Vertical displacements of numerical model at 29.6 kN [mm].

The orthotropic properties in the computational model were oriented according to the arrangement of the fibres of the upper and lower member (axis 1 according to Figure 7). In the first step of the analysis, the material properties were orthotropic with the mean value parameters taken from the design standard [8] for timber class GL24h: $E_{1}=11,500\;\mathrm{MPa}$, $E_{2}=E_{3}=300\;\mathrm{MPa}$, $\nu_{12}=\nu_{13}=\nu_{23}=0.35$ and $G_{12}=G_{13}=G_{23}=650\;\mathrm{MPa}$. The coefficient of friction μ was assumed to be 0 and, thus, frictionless. The force-displacement relation of the experiments showed non-linear behaviour at the beginning of the tests. Once the initial phase [6,7] was overcome, the connection could be seen as a perfect fit, resulting in a linear-elastic phase until a load of approximately 83 kN, corresponding to 72% of the averaged ultimate load. Subsequently, a loss of stiffness was noticeable until the ultimate load was reached, and the load began to drop. As mentioned before, a linear computational model only leads to a linear response of the structure, and progressive damage cannot be reproduced. Therefore, the numerical calculation was done for a 29.6 kN increment. Figure 8 and Figure 9 show displacement fields obtained from the numerical analysis at a force of 29.6 kN.

The stiffness *k* of the numerical model, defined as the ratio of 29.6 kN to the relative displacement of the extensometer points (see Figure 2 (right)), was equal to 90.08 kN/mm. This value indicated a poor approximation of the experimental results, of 72.1 kN/mm for specimen A_5 (the mean stiffness of the test series was 68.2 kN/mm), resulting in the decision of the authors to further calibrate the model using the results of the experiments.

### 4.2. Calibration of the Numerical Model According to the Results of the Experimental Investiagions

**Figure 10:**
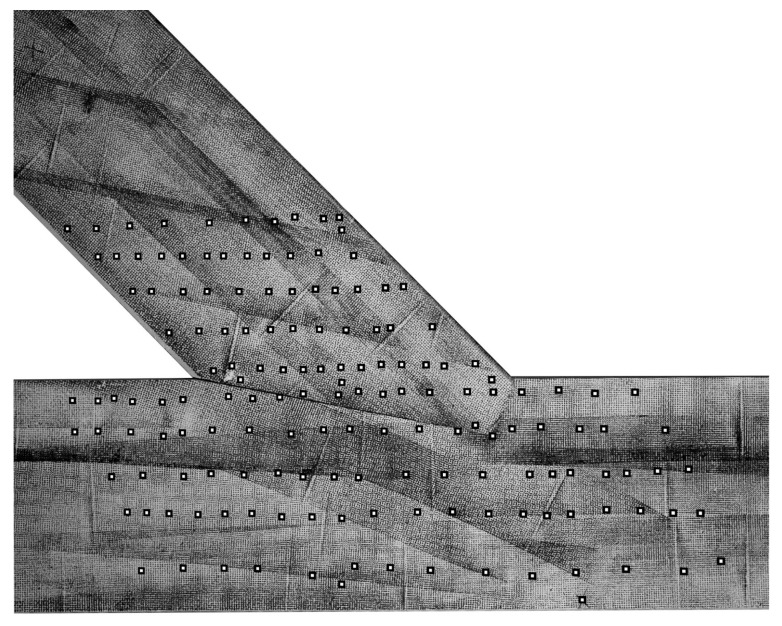
A total of 150 inspect points placed on the surface in the DIC post-processing to extract the displacements for the calibration of the numerical model.

The following material parameters were re-evaluated during the calibration of the linear-elastic model, with the parameters taken as variable: Young’s modulus, both in the longitudinal ($E_{1}$) and transversal $\left(E_{2}\right)$ direction, Kirchhoff’s modulus G_12_ and the friction factor μ. It was assumed that the geometry of the joint was modelled accurately and, therefore, geometrical factors were not used as calibration candidates. As already described in the previous section and clearly displayed in Figure 6, a load increment of 29.6 kN was chosen for the linear-elastic modelling, starting from a load of 40.5 kN up to a load of 70.1 kN. The vertical and horizontal displacements of 150 inspect points placed in the DIC post-processing, pictured in Figure 10, were used as reference points for the calibration.

**Figure 11:**
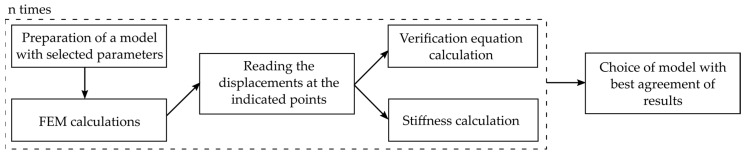
Flowchart of the calibration process.

During the calibration, with the process illustrated in Figure 11, 850 numerical models incorporating different parameters were automatically generated using Python and, subsequently, calculated using Abaqus. The displacement values of the defined inspect points were then evaluated using the previously programmed Python code with the implemented coordinates (see Figure 10) before being automatically transferred to an Excel file. In order to determine the most adequate parameters, resulting in a model matching the experimental results, a verification equation (Equation (3)) comparing the displacement fields was defined as:

(3) $$\|x\|\text{:}X,Y\to \mathbb{R},\;\|x\|=\sqrt{\overset{n}{\underset{i=1}{\sum }}{\left(Y_{i}-X_{i}\right)}^{2}}$$

where X is a set of 150 values taken from numerical model, and Y is the set of 150 values taken from the same places but from the experimental results seen in Figure 10. The calculation of the verification equation, Equation (3), was carried out automatically. With the calculation time of one FEA simulation equalling 84 s, the authors will consider using a more time-efficient approach in future works, for example, the Newton method or a meta-heuristic approach to reduce simulation time [12].

To compare the investigated models, three values were calculated for each: the horizontal ($\left\|x_{H}\right\|$) and vertical ($\left\|x_{V}\right\|$) displacement field according to the verification equation, Equation (3), and the stiffness *k* of the numerical model defined as the ratio of 29.6 kN to the relative displacement of the extensometer points (see Figure 2 (right)). The ranges of the parameter values, considered within the calibration, are listed in Table 1. 

**Figure 12:**
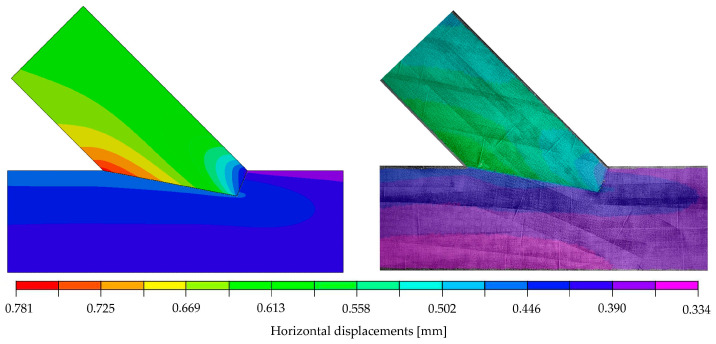
(**Left**): Horizontal displacements of the numerical model at a load of 29.6 kN; (**Right**): DIC-horizontal displacements on the surface of specimen A_5 at 70.1 kN with the initial picture at 40.5 kN (representing a load step of approximately 29.6 kN).

**Figure 13:**
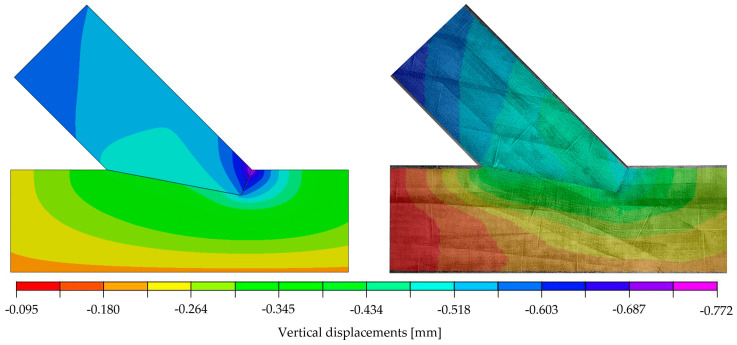
(**Left**): Vertical displacements of the numerical model at a load of 29.6 kN; (**Right**): DIC-vertical displacements on the surface of specimen A_5 at 70.1 kN with the initial picture at 40.5 kN (representing a load step of approximately 29.6 kN).

The calibration showed that the set of parameters which provided the best agreement of the numerical results and the laboratory test was: $E_{1}=10,350\;\mathrm{MPa}$, $E_{2}=196\;\mathrm{MPa}$, $\nu_{12}=0.35$, $G_{12}=728\;\mathrm{MPa}$ and μ=0.0. This model was characterised by the values $\|x_{H}\|=0.77753$, $\|x_{V}\|=0.77752\;$ and k=72.11 kN/mm. A very good compliance of the stiffness values from the numerical analysis (72.11 kN/mm) and the laboratory test (72.10 kN/mm) was achieved. Furthermore, it should be noted that, in the chosen model, very similar results from Equation (3), calculated for both directions, were obtained. Considering all results, the numerical modelling in the linear-elastic range using the orthotropic model showed an acceptable agreement with the experimental results in the linear-elastic range. The correspondence is clearly visible in Figure 12 and Figure 13, where the displacement fields obtained for the selected model are visualised and juxtaposed with those of the experiment. It should be emphasised that the surface conditions of the contact surface are to be seen as stochastic, as they are highly dependent on the accuracy of the samples and the material heterogeneity (e.g., twist of the fibres or small local knots in the timber).

The compliance of the experimental and numerical results obtained for the single-step joint in terms of stiffness and the verification equation, Equation (3), entitled the authors to conduct further computational simulations. In a subsequent step, a stress analysis was performed on the numerical model to determine possible forms of joint failure. A quantitative analysis of the calculated stresses was performed with the following mean values of strength parameters for spruce: tension parallel to the grain 30.00 MPa, compression parallel to the grain 32.00 MPa [24], compression perpendicular to the grain 3.57 MPa and shear 3.85 MPa [25]. The local failure mode was defined as a deformation associated with exceeding related strength parameters in the simulation. The linear computational model only allowed for the indication of the load at which the standard maximum stresses are exceeded. This state can be identified as the beginning of local failure, yet it does not necessarily lead to a load drop. Due to the assumptions made within the numerical model, progressive damage could not be considered.

With the numerical model being linear, it was possible to determine the load value at which a specified stress value exceeded the material strength. The determined load values indicating the location of extreme stress are listed in Table 2. The analysis thereof showed that the first to reach its maximum was shear (load approximately at 50 kN). If the load further increased to 52 kN in the numerical model, compressive failure perpendicular to the grain followed.

**Figure 14:**
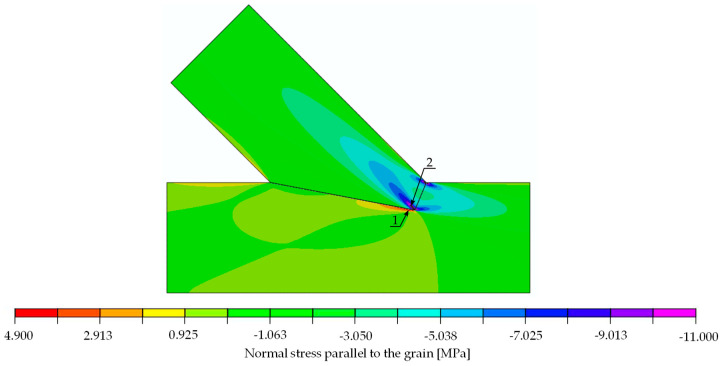
Normal stress parallel to the grain [MPa]. Force 29.6 kN.

**Figure 15:**
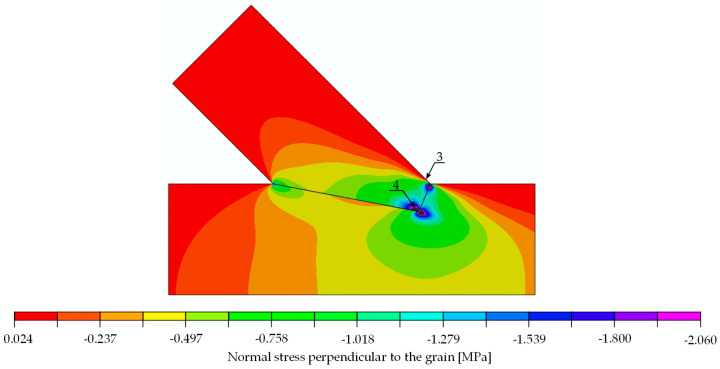
Normal stress perpendicular to the grain [MPa]. Force 29.6 kN.

**Figure 16:**
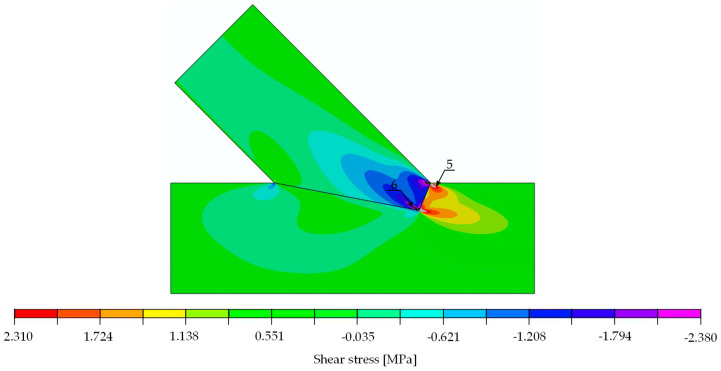
Shear stress [MPa]. Force 29.6 kN.

The stress distributions at a load of 29.6 kN are shown in Figure 14, Figure 15 and Figure 16. For the extreme values occurring in places of stress concentrations, the data were not extracted directly from that location but at a distance of 8 mm, allowing for reliable values close enough to the concentration point yet not obscured by the modelling.

Even though the linear model did not allow for the analysis of damage propagation, and the indicated values did not coincide with load-bearing capacity obtained during experimental results, comparing load values taken from the numerical model with those from the experiments showed that an exceedance of the strength values is not necessarily accompanied by a decrease in the joints’ stiffness, as seen in Figure 6. In order to correctly represent the load-bearing capacity, a failure criterion had to be introduced to the numerical model.

**Figure 17:**
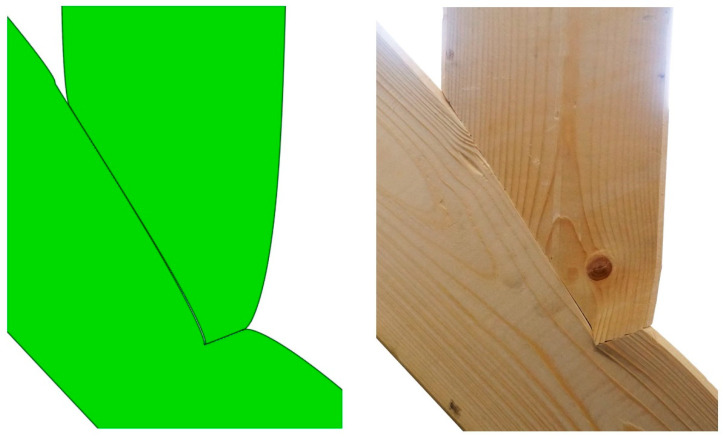
(**Left**): Deformation of numerical model; (**Right**): failure of single-step joint in the experiment.

When looking at the connection itself, Figure 17 shows a comparison of the joint post loading for both the simulation and the experiment. The upper beam layers separated and shifted relative to each other at the connection point, indicating that the allowable shear stress had been exceeded. In addition, a bending of the aforementioned fibres was noticeable, most likely caused by exceeding the allowable compressive stresses along the fibres. The numerical model indicated that the latter occurs at a load force of 87 kN. The value varied from the actual results due to possible redistributions associated with earlier strength attainment in other directions. Even though the exact failure load could not be calculated using the model, valuable insights regarding failure type could be derived, as is clearly visible in Figure 17, where the joint deformation in both physical and numerical models were in good compliance.

## 5. Application of the Calibrated Numerical Model on a Double-Step Joint

**Figure 18:**
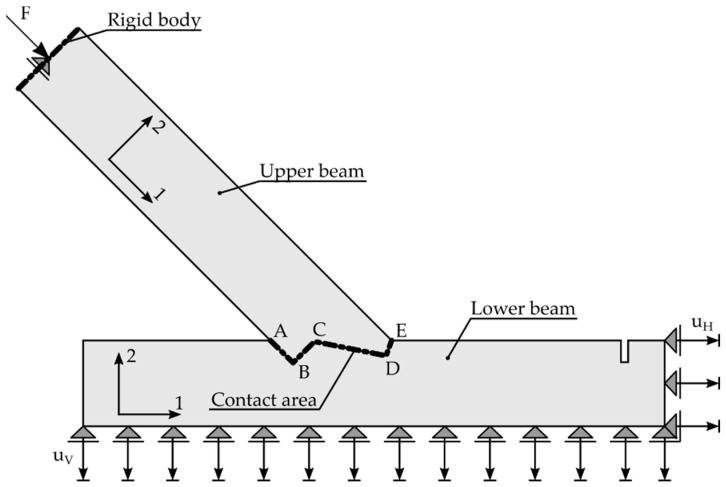
Geometrical data of the FEM model for the double-step joint. Axis 1 represents the orientation parallel to the grain.

The main goal of the authors for the computational analysis was to create a model that could also be applied to other geometries. The results of experimental investigations of double-step joints (Series B) were used to validate the calibrated model, to predict the stiffness and indicate the possible failure mode of the new connection. The geometry of the joint, load introduction point and the geometrical boundary conditions are presented in Figure 18 and can also be found in [1].

**Figure 19:**
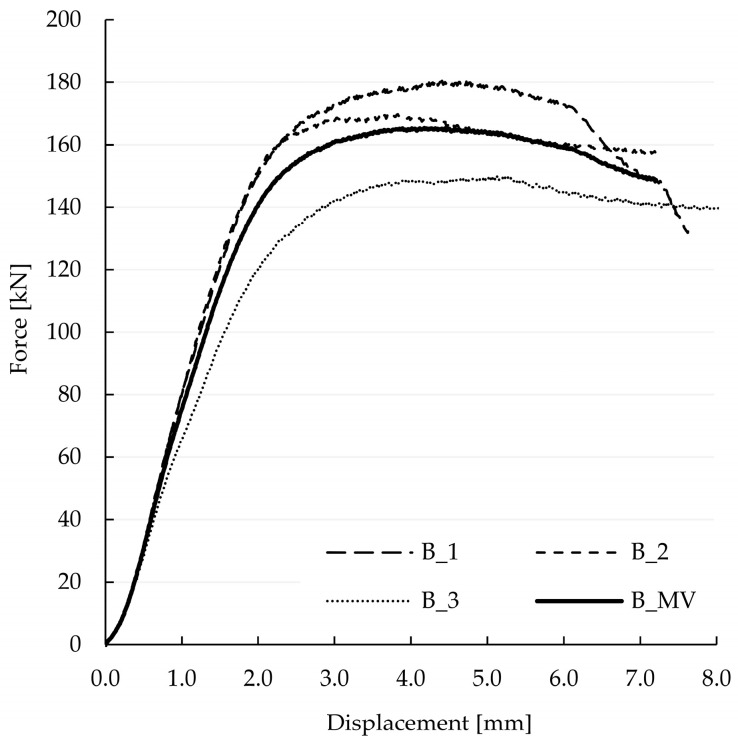
Force-displacement curves of all three single-step specimens, as well as the mean value MV of the three specimens.

The results of the experimental investigation of Series B are presented in Figure 19 in form of a force-displacement diagram, serving as a reference for the validation of the predicted stiffness. The test series consisted of three specimens tested under the same conditions as Series A. Furthermore, the specimens were produced out of the same material.

**Figure 20:**
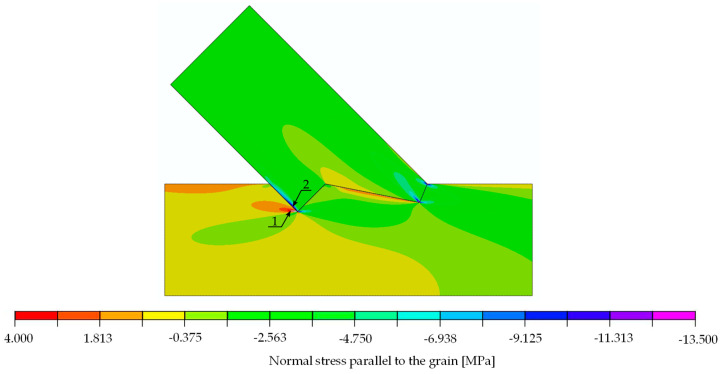
Double-step joint FEM modelling—normal stress parallel to the grain [MPa]. Force 29.6 kN.

**Figure 21:**
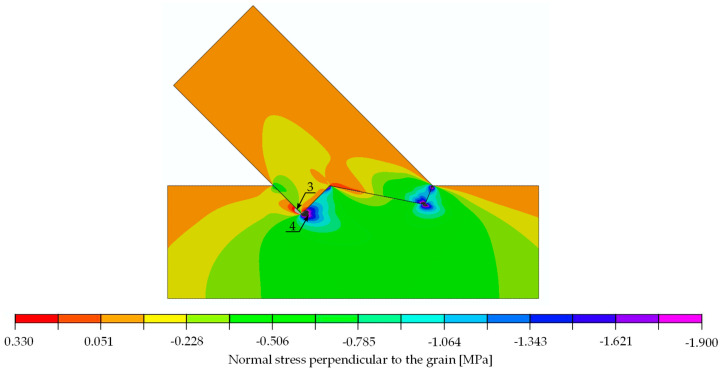
Double-step joint FEM modelling—normal stress perpendicular to the grain [MPa]. Force 29.6 kN.

**Figure 22:**
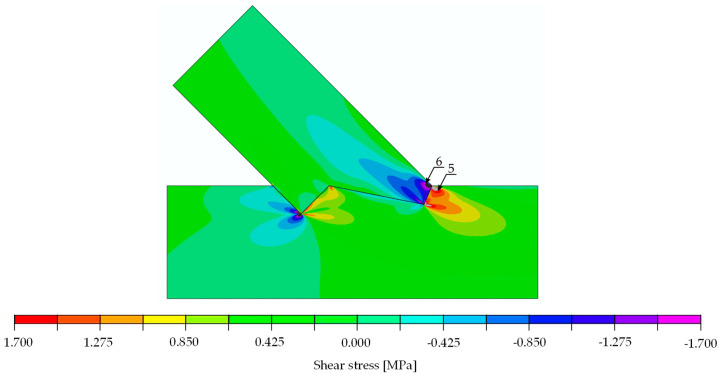
Double-step joint FEM modelling—shear stress [MPa]. Force 30 kN.

The results of the calculations in form of stress distribution and the location where stress equalled material strength (according to Table 3 for the double-step joints) can be seen in Figure 20 (normal stress parallel to the grain), Figure 21 (normal stress perpendicular to the grain) and Figure 22 (shear stress).

The stress values at a load of 29.6 kN, as well as the locations and load values at which the material properties were exceeded, are listed in Table 3 for the double-step joint.

**Figure 23:**
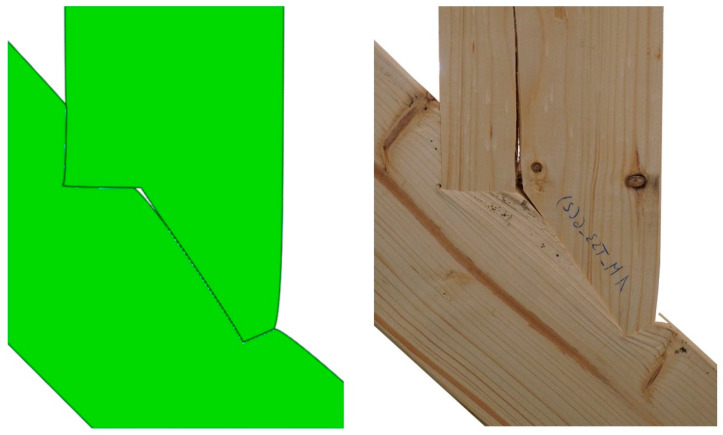
(**Left**): Deformation of numerical model; (**Right**): failure of double-step joint in the experiment.

**Figure 24:**
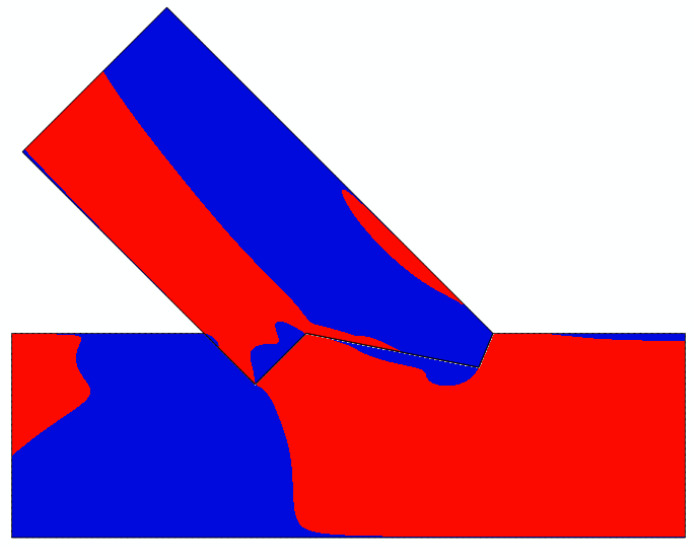
Shear stress distribution: negative (blue) and positive values (red).

The analysis of Figure 20 through Figure 22 and Table 3 allowed for the prediction of possible local failure modes. Based on the calculations of the single-step joint, the numerical model for the double-step joint was loaded with 29.6 kN. This assumption can be considered correct as the linear range of the specimens starting at 12 kN and ending at 79 kN, as pictured in Figure 19. According to the linear model, the first stress limit reached was that of tensile strength perpendicular to the grain close to point B (see Figure 18) at a load of 45.5 kN, followed by a failure mode related to exceeding the shear stress limit close to point E. The last-mentioned mode is visualised in Figure 23, where the timber layers on the right-hand side of the connection separated and moved relative to each other. The crack visible in Figure 23 was further pre-announced by the distribution of shear stress (shown in Figure 24), where the sign changed within the upper beam (which is related to the change of the direction of shear deformation), suggesting the possibility of a crack occurrence starting at point B in the upper beam. 

It should be noted that, according to [6], each analysed specimen of the double-step joint exhibited a slightly different behaviour in the experimental tests, in terms of both the stiffness and the failure modes. During the testing of some samples, the failure modes observed in the numerical analysis occurred nearly simultaneously within the experiments. Furthermore, it should be highlighted that the double-step joint has a higher sensitivity to manufacturing inaccuracies and randomness of the mechanical properties of the wood due to its design. Even though it is not possible to create a general valid numerical model that can accurately predict failure modes of such connections, it was shown within the double-step joint analysis that the proposed linear model could be used to give a general indication of possible damage modes and locations.

Moreover, the calibrated linear model allows for a quantification of the stiffness of the double-step connection. The stiffness k, obtained from the numerical analysis, was calculated to 86.0 kN/mm, while that of the Series B specimens equalled 87.0 kN/mm. The good compliance of the results shows the viability of the proposed linear numerical model in regard to first assessments of timber–timber connections.

## 6. Concluding Remarks

The authors presented a simplified strategy using a linear-elastic model of an orthotropic body for the numerical simulation of single- and a double-step timber joints under compression. The numerical model was calibrated using the results of experimental investigations on a series of single-step joints. A DIC measurement system allowed for the documentation of the displacements on the surface of the specimens, which made it possible to quantify their rigid movement and deformation. Due to the fact that numerical results obtained with the assumption of material parameters taken from design code did not reflect laboratory tests to a satisfactory degree, the results obtained for the single-step joint were used for the calibration of the FE model with the use of Abaqus software and Python codes, resulting in a calibrated model without specified material parameters. 

The presented numerical and experimental analyses were qualitatively consistent and confirm the applicability of the approach. The applied numerical model made it possible to satisfactorily quantify the stiffness of both connections (inclination of the linear-elastic phase in the force-displacement diagrams) and to predict the location of local failures. However, in the single-step joint, the prediction was more accurate, based on the fact that the model was calibrated for this type of joint, and the connection itself is less prone to inaccuracies of the geometry. The possible local failure modes were identified in places where strength limits were exceeded. However, it should be highlighted that such states do not necessarily lead to a load drop—they only indicate that some local damages occur.

The main advantage of a linear analysis is the speed of calculation and the relative ease of model building. However, interpretation of the results requires knowledge of the mechanical behaviour of timber. The proposed model can be extended by introducing failure criteria such as the Tsai–Wu criterion mentioned in [26]. This would allow for the prediction of the load-bearing capacity of the joint and indicate the failure modes and stiffness of the system. However, one should keep in mind the heterogeneity of the material, which introduces uncertainty in the calculations. According to the authors, crack mechanics modelling of the development of the destruction zone is not advisable if the modelling and computational effort should be kept to a certain boundary due to the scattering of wood characteristics. Inconsistencies of the samples, such as the arrangement of fibres, the presence of small knots and heterogeneity, as well as the inevitable geometrical inaccuracies in the fabrication of samples, should, therefore, be considered.

The proposed numerical model is recommended for future, non-destructive research of timber connections to estimate the stiffness and the failure mode. Possible future directions of research could be related to the analysis of various geometries of joints, including gaps between the connected beams, and optimisation of contact geometry in order to avoid stress concentrations and failure analysis in the frame of crack mechanics.

## Figures and Tables

**Table 1:** Range of parameter values *E*_1_, *E*_2_, G_12_ and μ.

Parameter	Min	Max
*E*_1_ [MPa]	100	12,650
*E*_2_ [MPa]	10	400
G_12_ [MPa]	50	1000
μ [-]	0.0	0.4

**Table 2:** Location and values of extreme values of stresses in single-step joint with the information of load at which those values reach respective limits.

Stress		Value for 30 kN [MPa]	Strength [MPa]	Limit Load [kN]	Location: Point (Beam)	Figure
parallel to the grain	tension	4.900	30.00	183.7	1 (lower)	Figure 14
	compression	11.000	32.00	87.3	2 (upper)	Figure 14
perpendicular to the grain	tension	0.024	0.50	625.0	3 (upper)	Figure 15
	compression	2.060	3.57	52.0	4 (upper)	Figure 15
shear	positive	2.310	3.85	50.0	5 (lower)	Figure 16
	negative	2.380	3.85	48.5	6 (upper)	Figure 16

**Table 3:** Stress values and location at a load of 29.6 kN for the double-step joint including the calculation of the limit load for the individual stress (considering the linear progression).

Stress		Value for 29.6 kN [MPa]	Strength [MPa]	Limit Load [kN]	Location: Point (Beam)	Figure
parallel to the grain	tension	4.00	30.00	225.0	1 (lower)	Figure 20
	compression	13.49	32.00	71.2	2 (upper)	Figure 20
perpendicular to the grain	tension	0.33	0.50	45.5	3 (lower)	Figure 21
	compression	1.86	3.57	57.6	4 (lower)	Figure 21
shear	positive	1.73	3.85	66.8	5 (lower)	Figure 22
	negative	1.71	3.85	67.5	6 (upper)	Figure 22
